# The Ameliorative Effects of Isorhynchophylline on Morphine Dependence Are Mediated Through the Microbiota-Gut-Brain Axis

**DOI:** 10.3389/fphar.2021.526923

**Published:** 2021-06-08

**Authors:** Zhu Chen, Chen Zhijie, Zhou Yuting, Li Chan, Xiao Shilin, Zhou Qichun, Ou Jinying, Li Jing, Luo Chaohua, Mo Zhixian

**Affiliations:** ^1^ School of Traditional Chinese Medicine, Southern Medical University, Guangzhou, China; ^2^ The Second Affiliated Hospital of Guangzhou University of Chinese Medicine, Guangzhou, China; ^3^ Central Laboratory, Southern Medical University, Guangzhou, China

**Keywords:** gut microbiota, zebrafish, morphine, isorhynchophylline, antibiotic

## Abstract

Morphine abuse is a global public health problem. Increasing evidence has shown that gut microbiota dysbiosis plays an important role in several central nervous system diseases. However, whether there is an association between gut microbiota and morphine dependence remains unclear. In this study, the effects of isorhynchophylline on morphine dependence were evaluated based on the microbiota-gut-brain axis (MGBA). The results showed that isorhynchophylline could reverse the changes in alpha and beta diversity, composition, and richness of the intestinal flora occurring in morphine-dependent zebrafish, as well as the morphine-induced changes in the expression of MGBA-related genes in BV2 cells and the brain and intestine of zebrafish. Based on the results, we then used antibiotics to evaluate whether disrupting the gut microbiota would affect morphine addiction in zebrafish. The results showed that the antibiotic-induced intestinal floral imbalance changed the behavior of morphine-dependent zebrafish, the characteristics of the zebrafish intestinal flora, and the expression of MGBA-related genes in the zebrafish brain and intestine. Importantly, we also show that, following antibiotic administration, the ameliorative effects of isorhynchophylline on morphine addiction were lost. Together, our results indicate that the gut microbiota interacts with the brain, and dysbiosis of the intestinal flora may affect the efficacy of isorhynchophylline in the body. Our findings provide a novel framework for understanding the mechanisms of morphine addiction through the MGBA and may provide new therapeutic strategies for the use of Chinese medicines in the prevention of drug addiction.

## Introduction

Morphine is a potent opioid analgesic that is also highly addictive, and its abuse is a serious social and public health concern worldwide. Long-term morphine use results not only in serious damage to the central nervous system (CNS), but also commonly leads to opioid-induced bowel dysfunction (OBD) such as nausea, vomiting, gastroesophageal reflux, and constipation (Brock et al., 2012). Currently, investigation of the mechanisms involved in morphine addiction is primarily concentrated on the CNS; however, this has its limitations, and new strategies to investigate the mechanisms involved in morphine addiction are required.

Traditional Chinese medicine (TCM) is commonly used to treat addiction, presenting multitarget curative potential and nonaddictive properties. Isorhynchophylline is one of the main active ingredients of *Uncaria rhynchophylla*, a herb used in TCM that has been shown to protect against cerebral ischemia and nerve cell damage, and also exhibits anti-inflammatory properties (Kang et al., 2002; Yuan et al., 2009; Lu et al., 2012). Recent studies have shown that isorhynchophylline exerts important pharmacological effects on the nervous system in mice, such as improving cognitive and memory impairment, antagonizing Aβ-induced neurotoxicity, and eliciting antidepressive effects (Xian et al., 2013; Xian et al., 2014a; Xian et al., 2014b; Xian et al., 2017).

The human intestinal microflora is a complex and large ecosystem. The total number of microorganisms that comprise the intestinal flora has been estimated at approximately three times the total number of human cells. Gut microorganisms have a symbiotic relationship with their host and help maintain the host’s physiological homeostasis (Sleator, 2010). De Palma named the interaction between gut microbiota (GM) and the brain the “microbiota-gut-brain axis” (MGBA), which includes endocrine, neural, immune, and metabolic pathways (De Palma et al., 2014). Studies have shown that germ-free (GF) mice display increased motor activity and reduced anxiety-like behavior when compared with specific-pathogen-free (SPF) mice (Diaz et al., 2011; Zeng et al., 2016). Moreover, oral administration of the human commensal *Bacteroides fragilis* was shown to improve intestinal permeability, GM composition, and autism spectrum disorder (ASD)-related communication, anxiety-like and sensory-moter behavior, and stereotyping in the offspring of maternal immune activation (MIA) model mice (Hsiao et al., 2013). Studies have also shown that Alzheimer’s disease (AD) patients have significantly lower GM diversity than people without AD, while a significant correlation exists between changes in GM abundance and the clinical severity of this disease (Liu et al., 2019).

Based on a new understanding of the MGBA, we hypothesized that GM dysbiosis may be a contributing factor to the development of drug addiction. In this study, therefore, we investigated the interaction between GM and the brain in morphine dependence using zebrafish as a model, and discuss the effects of isorhynchophylline in terms of immune- and CNS-related pathways. This study provided new ideas for the development of novel diagnostic and therapeutic strategies for the treatment of drug addiction.

## Materials and Methods

### Cell Culture and Grouping

**FIGURE1:**
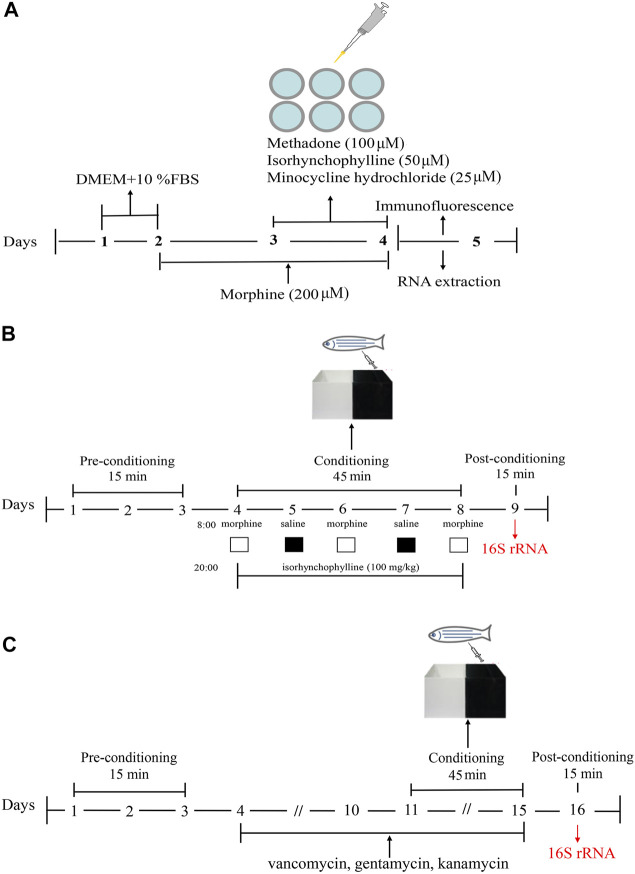
Schematic of the experimental design. **(A)** Schematic of the experimental design for analysis of morphine-induced activation of BV2 cells. **(B)** Schematic of the experimental design for the CPP test. **(C)** Schematic of the experimental design for antibiotic treatment combined with the CPP test.

BV2 cells were maintained in high glucose Dulbecco’s modified Eagle’s medium (DMEM) supplemented with 10% fetal bovine serum (FBS) and 1% penicillin-streptomycin. Cells were divided into the following five treatment groups: control (C), morphine (200 μM, M), morphine + methadone (100 μM, M + M), morphine + isorhynchophylline (50 μM, M + I), and morphine + minocycline hydrochloride (25 μM, M + MH). As shown in the flowchart in Figure 1A, after 24 h of BV2 cell culture, morphine (200 µM) was administered to the cells for 24 h in addition to C, followed by co-administration with methadone, isorhynchophylline, and minocycline hydrochloride for 24 h.

### Animals

Adult zebrafish (AB strain, male and female in half, weight: 0.3–0.5 g, age: 6–10 month-old) were provided by the Southern Medical University. Standard fish care and maintenance protocols were followed carefully. Environmental variance was kept to a minimum for all behavioral experiments. Adult zebrafish were maintained in tap water on a photoperiod with 14 h of light (8:30 am–10:30 pm) and 10 h of dark, and were fed twice daily with flake food.

### Drugs

Morphine was provided by the PLA General Logistics Department supply station (710303). Methadone (raw material powder), was bought from Tianjin Central Pharmaceutical Co., Ltd., (020111). Isorhynchophylline was purchased from Jiangxi Baicaoyuan Biotechnology Co., Ltd. (BCY-0778). Tricaine methanesulfonate (MS222), was purchased from Sigma Aldrich.

### Conditional Position Preference (CPP) in Zebrafish

The CPP test was performed using a previously published protocol (Zhu et al., 2017). A CPP box is a tank with a length of 16 cm, a width of 9 cm, and a height of 9 cm. A transparent movable baffle is inserted in the middle to divide the box into two compartments, one of which is painted black and the other is transparent. The CPP test consisted of three phases and lasted for 9 days **(** Figure 1B). In the first stage (days 1–3), the zebrafish were placed in the CPP box for 2 days of adaptive feeding, following which the activity of the zebrafish in the CPP box was recorded for 15 min. As the results showed that the black compartment was preferred by 95% of the zebrafish, the black compartment was selected as the preference compartment, and the transparent compartment was selected as the drug-paired compartment. Excluding the zebrafish that showed an obvious preference for the transparent compartment, the zebrafish were randomly divided into the following four treatment groups: control (C), morphine (40 mg/g, M), morphine + methadone (40 mg/g, M + M), and isorhynchophylline (100 mg/g, M + I). Stage 2 lasted from day 4 to day 8. On days 4, 6, and 8, at 08:00, the zebrafish in all the treatment groups (except for the C group) were injected intraperitoneally with morphine, and then placed in the drug-paired box for 45 min. On days 5 and 7 at 08:00, the zebrafish in all the groups were injected intraperitoneally with an equal volume of saline, and then placed in the CPP box for 45 min. On days 4–8, at 20:00, the fish in the control group and morphine-treatment group were injected intraperitoneally with saline, while the other treatment groups were administered the corresponding drugs. In Stage 3 (day 9), 24 h after the last morphine administration, the CPP test was performed for 15 min to record the CPP of the zebrafish. Noldus animal behavior analysis system (Ethovision XT 8.5) was used to track zebrafish movement. The experimenters are blinded to the treatment groups.

### Antibiotic Treatment Combined With the CPP Test

The zebrafish were randomly divided into the following five treatment groups: control (C), antibiotic (A), antibiotic + morphine (A + M), antibiotic + morphine + methadone (A + M + M), and antibiotic + morphine + isorhynchophylline (A + M + I). This section consisted of four stages lasting for 16 days (Figure 1C). The first stage (days 1–3) consisted of zebrafish adaptive feeding and preconditioning for the CPP test. The second stage (days 4–10) consisted of antibiotic pretreatment. All the treatment groups (except the control group) were fed in system water containing vancomycin (100 mg/L), gentamicin (10 mg/L), and kanamycin (5 mg/L) for 1 week. The third stage (days 11–15) comprised the conditioning phase, which was as described above, except for the last stage (day 16), when the postconditioning CPP test was performed on the zebrafish. Noldus animal behavior analysis system (Ethovision XT 8.5) was used to track zebrafish movement. The experimenters are blinded to the treatment groups.

### Collection of Brain Tissue

After the test of CPP, adult zebrafish were anesthetized in tricaine and killed in iced water. Following removal of the skull and exposure of the brains, zebrafish were decapitated and the brain was taken out under the microscope, and store at −80°C.

### Collection of the Intestine and Its Contents

The zebrafish were frozen to death, the body surface was wiped with 75% alcohol, the abdomen was cut with sterile dissecting scissors, the intestinal organs were taken out, the outer wall of the intestine was washed with PBS, and the surface was dried with absorbent paper, and stored at −80°C.

Take out the entire intestine in a sterile state, cut off the contents of the intestine with sterile dissecting scissors, and store at −80°C.

### Immunofluorescence Observation of Morphine-Induced BV2 Cell Activation

To determine the expression and localization of allograft inflammatory factor 1(AIF1) in BV2 cells, double immunocytochemistry staining was performed. Briefly, BV2 cells were fixed in 4% paraformaldehyde, permeabilized with 0.5% Triton X-100, blocked with 3% BSA, and incubated overnight with a primary antibody against AIF1 (1:100, Abcam) at 4°C. The following day, the cells were then incubated with Alexa Flour 488 goat anti-rabbit IgG (H + L) antibody in the dark at room temperature for 5 min, then stained with DAPI, followed by three washes with PBS. Fluorescence images were obtained under an inverted fluorescence microscope.

### 16s rRNA Sequencing Analysis

Microbial DNA was extracted from the zebrafish gut using the E. Z.N.A.® soil DNA Kit (Omega Bio-tek, Norcross, GA, United States) according to the manufacturer’s protocol. The final DNA concentration and DNA purity were determined using a NanoDrop 2000 UV-vis spectrophotometer, and DNA quality was further evaluated by 1% agarose gel electrophoresis. The V3-V4 hypervariable regions of the bacterial 16S rRNA gene were amplified with primers 338F (5′-ACT​CCT​ACG​GGA​GGC​AGC​AG-3′) and 806R (5′-GGACTACHVGGGTWTCTAAT-3′) using a thermocycler PCR system (GeneAmp 9700, ABI, United States). The PCR conditions and system are shown in Supplementary Tables S1,S2.

Purified amplicons were pooled in equimolar concentrations and paired-end sequenced (2 × 300) on an Illumina MiSeq platform (Illumina, San Diego, CA, United States) according to the standard protocols of the Majorbio Bio-Pharm Technology Co., Ltd (Shanghai, China).

Raw fastq files were demultiplexed, quality-filtered by Trimmomatic, and merged by FLASH. Operational taxonomic units (OTUs) were clustered with a 97% similarity cutoff using UPARSE (version 7.1 http://drive5.com/uparse/) and chimeric sequences were identified and removed using UCHIME. The taxonomic status of each 16S rRNA gene sequence was analyzed by RDP Classifier algorithm (http://rdp.cme.msu.edu/) against the Silva (Silva 128/16s-bacteria) 16S rRNA database using a 70% confidence threshold.

### Quantitative Real-Time PCR

Total RNA was extracted from BV2 cells and the brain and gut of zebrafish using trizol reagent (Takara, Dalian, China) as previously described (Fang et al., 2017), and reversed-transcribed into cDNA using a primerScript RT Reagent Kit (Takara). qPCR was performed on a Light Cycler 96 system (Roche, Germany) using the TB Green premix Ex Taq Reagent Kit (Takara), according to the manufacturer’s recommendations. The primers used in this experiment were designed by Ribobio Co., Ltd (Guangzhou, China) and are listed in Supplementary Table S3.

### Statistical Analysis

Values are expressed as mean ± SD. All data were analyzed using one-way analysis of variance (ANOVA), followed by the least significant difference (LSD) post hoc test (twotailed). All statistical analyses were performed using SPSS software (version19.0). *p* < 0.05 was considered to be statistically significant.

## Results

### Isorhynchophylline Inhibits Morphine-Induced Activation of AIF1 Expression in BV2 Cells

**FIGURE 2:**
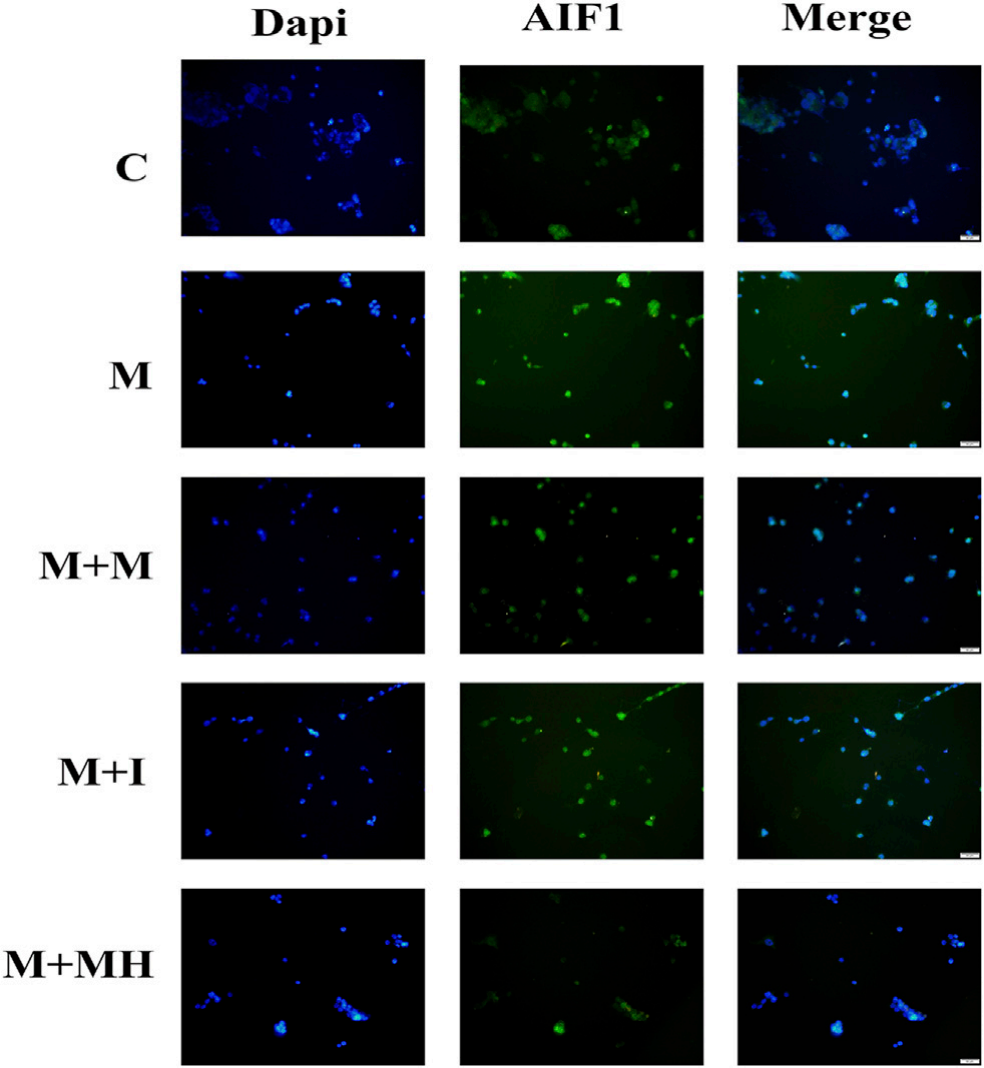
Cells were subjected to immunofluorescence analysis using an antibody directed against AIF1 (green). Nuclei were counterstained with DAPI (blue). The fluorescence was visualized and imaged under a fluorescence microscope. C: control group; M: morphine group; M + M: morphine+methadone group; M + I: morphine+isorhynchophylline; M + MH: morphine+minocycline hydrochloride.

AIF1 is an activation marker protein of microglia and is often used to evaluate the activation of microglia (Walter and Crews, 2017). When microglia are activated by exogenous substances, AIF1 expression is up-regulated. To confirm the effects of morphine and isorhynchophylline exposure on the activation status of BV2 cells, we analyzed the expression of AIF1 by immunofluorescence. The results showed that morphine treatment led to the activation of BV2 cells, whereas exposure to isorhynchophylline attenuated the morphine-induced activation of AIF1 in BV2 cells (Figure 2).

### qPCR Analysis of Relative mRNA Expression Levels in C-, M-, M + M-, M + I-, and M + MH-Treated BV2 Cells.

**FIGURE 3:**
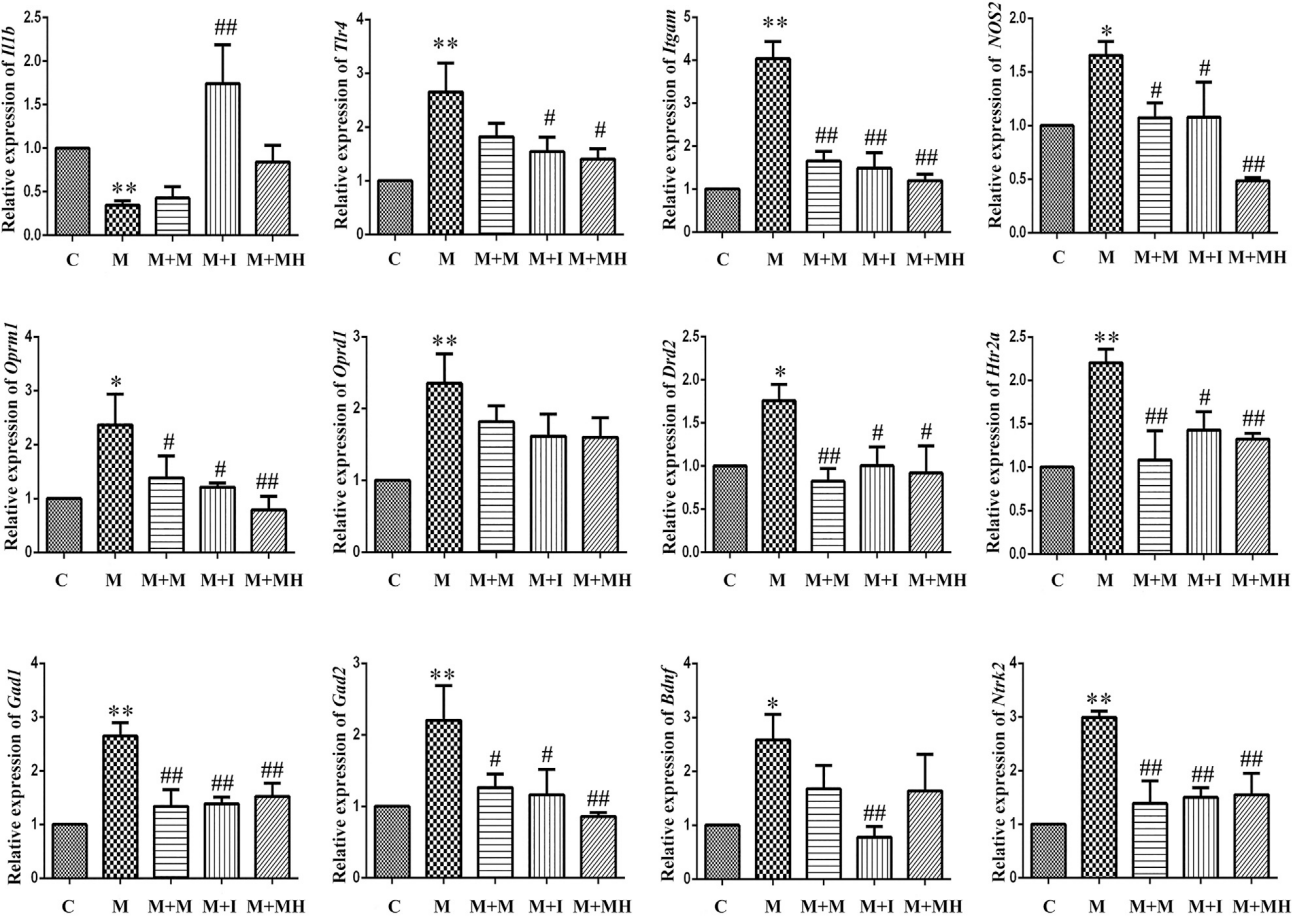
qPCR analysis of *Il1b*, *Tlr4*, *Itgam*, *Nos2*, *Oprm1*, *Oprd1*, *Drd2*, *Htr2a*, *Gad1*, *Gad2*, *Bdnf*, and *Ntrk2* expression in BV2 cells (*n* = 3). *p < 0.05, **p < 0.01 *vs* the C group; ^#^ *p* < 0.05, ^##^ *p* < 0.01 *vs* the M group. C: control group; M: morphine group; M + M: morphine + methadone group; M + I: morphine + isorhynchophylline group; M + MH: morphine + minocycline hydrochloride group.

The changes in mRNA expression in BV2 cells are shown in Figure 3. Compared with the control group, the mRNA expression of interleukin 1 beta (*Il1b*) in BV2 cells treated with morphine was significantly decreased, whereas the mRNA expression of *Il1b* in the M + I group was significantly increased compared with that of the M group. Compared with the control group, the mRNA expression levels of toll like receptor 4 (*Tlr4*), integrin subunit alpha M (*Itgam*), and nitric oxide synthase 2 (*Nos2*) in the M treatment group were significantly decreased, whereas the mRNA expression levels of *Tlr4*, *Itgam*, and *Nos2* in the M + I and M + MH groups were significantly higher than those in the M group. Compared with the control group, the mRNA expression of opioid receptor mu 1 (*Oprm1*) and opioid receptor delta 1 (*Oprd1*) in the M group was significantly decreased, whereas the mRNA expression level of *Oprm1* in the M + I and M + MH groups was significantly increased compared with that of the M group. Compared with the control group, the mRNA expression levels of dopamine receptor D2 (*Drd2*), 5-hydroxytryptamine receptor 2A (*Htr2a*), glutamate decarboxylase 1 (*Gad1*), and *Gad2* were significantly higher in the M group than in the control group, whereas the mRNA expression levels of *Drd2*, *Htr1aa*, *Gad1*, and *Gad2* in the M + I and M + MH groups were significantly reduced when compared with those in the M group. Compared with the control group, the mRNA expression levels of brain derived neurotrophic factor (*Bdnf*) and neurotrophic receptor tyrosine kinase 2 (*Ntrk2/Trkb*) in the M group were significantly increased, whereas the mRNA expression levels of *Bdnf* and *Ntrk2* in the M + I and M + MH groups were significantly lower than those in the M group.

### Isorhynchophylline Inhibits Morphine-Induced CPP Responses

**FIGURE 4:**
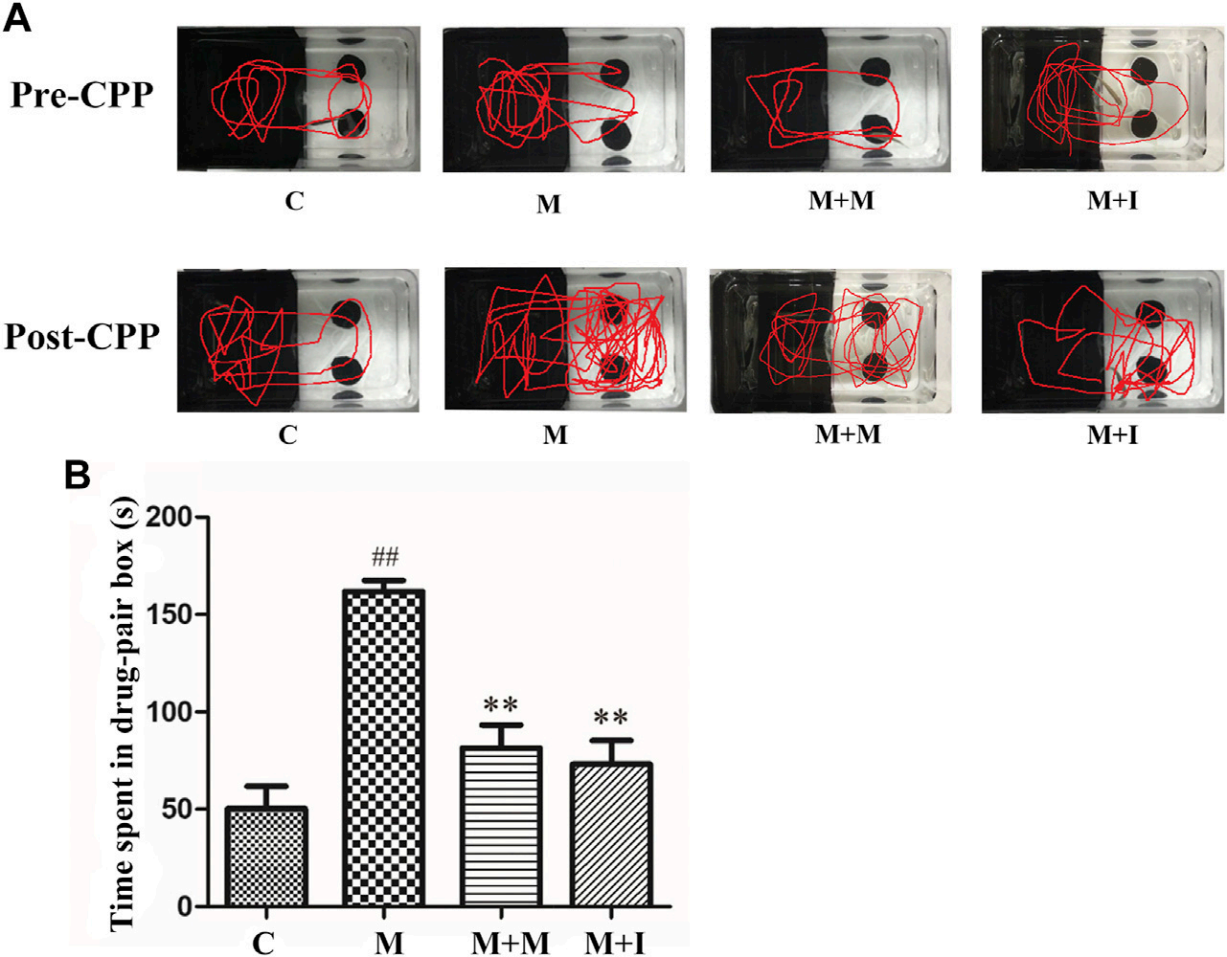
Isorhynchophylline can inhibit morphine-induced CPP responses. **(A)** Road maps of zebrafish in the CPP compartment. **(B)** Time spent in the drug-paired box post-CPP (*n* = 8). *p < 0.05, **p < 0.01 *vs* the C group; ^#^ *p* < 0.05, ^##^ *p* < 0.01 *vs* the M group. C: control group; M: morphine group; M + M: morphine + methadone group; M + I: morphine + isorhynchophylline group.

We performed a CPP test on zebrafish to study the effects of isorhynchophylline on morphine-induced CPP responses. As shown in Figure 4, compared with the control group, the resident time of zebrafish from the M group in the drug-paired compartment was significantly increased (*p* < 0.01), whereas the resident time of zebrafish from the M + I group in the white box was markedly lower than that of the M group (*p* < 0.01).

### Isorhynchophylline Ameliorates the Morphine-Induced Changes in Zebrafish Gut Microbiota Composition

**FIGURE 5:**
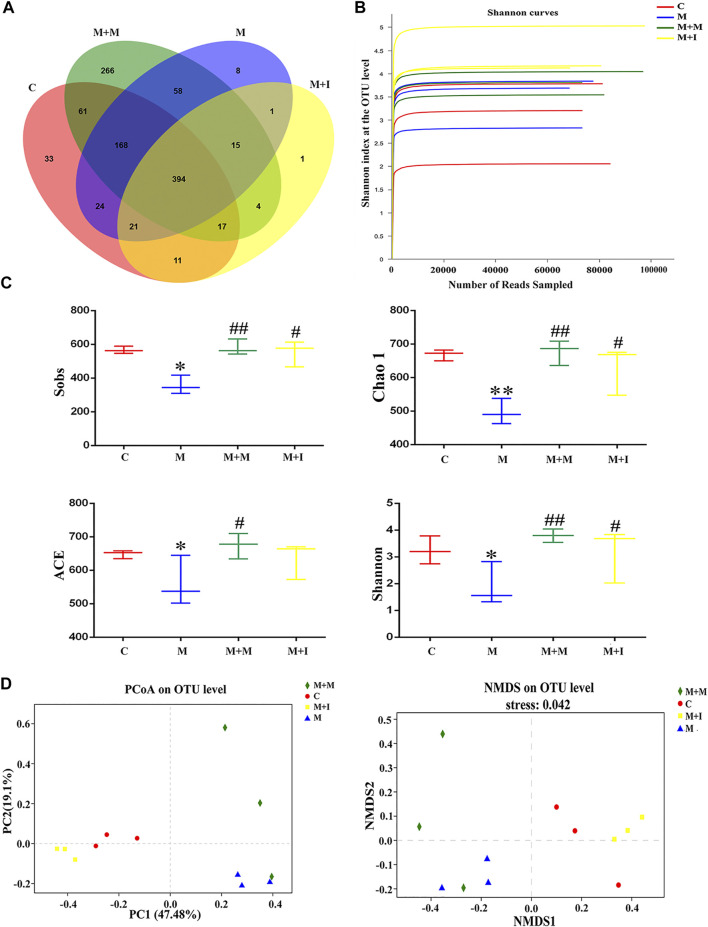
Comparison of community structure among the fecal microbiota of the C, M, M + M, and M + I groups. **(A)** Venn diagram illustrating the overlap of the operational taxonomic units (OTUs) identified in the fecal microbiota of the four groups. **(B)** Shannon curves among the four groups. **(C)** Alpha diversity of the fecal microbiome among the four groups according to Sobs, Chao, ACE, and Shannon indices. **(D)** Beta diversity of the fecal microbiome among the four groups based on principal coordinate analysis (PCoA) and nonmetric multidimensional scaling (NMDS). *p < 0.05, **p < 0.01 *vs* the C group; ^#^ *p* < 0.05, ^##^ *p* < 0.01 *vs* the M group. C: control group; M: morphine group; M + M: morphine + methadone group; M + I: morphine + isorhynchophylline group.

To assess morphine-induced changes in the gut microbiome of zebrafish, we performed massively parallel sequencing using the Miseq platform. We obtained 900,777 optimized sequences in all samples with an average length of 439.07 bp. As shown by the Venn diagram (Figure 5A), a total of 1,082 operational taxonomic units (OTUs) were identified among all the samples, 394 of which were shared by the four groups. A total of 33 OTUs were unique to the control group, eight to the M group, 266 to the M + M group, and one to the M + I group. The Shannon curve was saturated, indicating that sufficient sequencing depth was obtained (Figure 5B). The community richness and diversity of the M group were significantly decreased, as indicated by the reduced Sobs, Chao 1, ACE, and Shannon index values (Figure 5C). Distance matrices (beta diversity) between samples were generated based on Bray–Curtis similarity algorithms at the OTU level, and reported according to principal coordinate analysis (PCoA) and nonmetric multidimensional scaling (NMDS) (Figure 5D). The PCoA and NMDS results showed that were differences in microbial profiles among the four groups (C, M, M + M, and M + I).

### Isorhynchophylline can Ameliorate the Morphine Dependence-Induced Changes in the Gut Microbiota of Zebrafish

**FIGURE 6:**
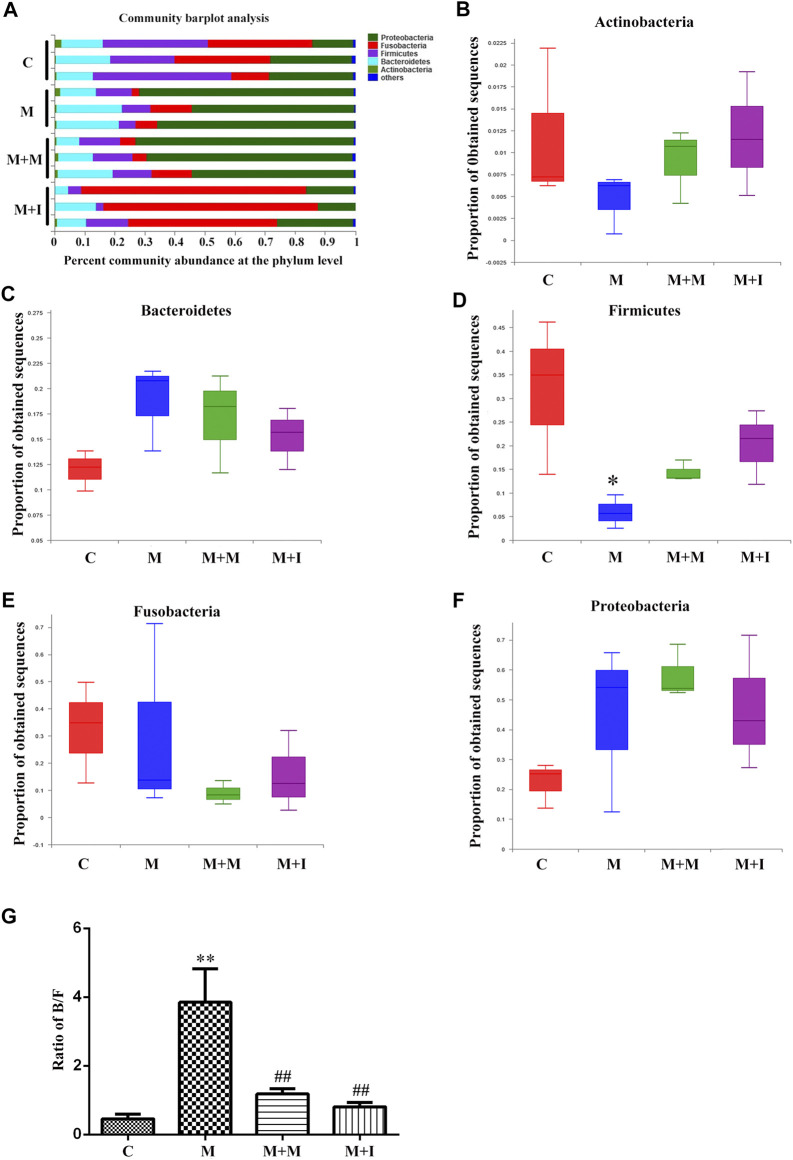
Comparison of species composition among the fecal microbiota of the C, M, M + M, and M + I groups at the phylum level. **(A)** Relative abundances of the phyla present in samples from the four groups. Percent abundance of **(B)** Actinobacteria **(C)** Bacteroidetes **(D)** Firmicutes **(E)** Fusobacteria, and **(F)** Proteobacteria at the phylum level among the four groups. **(G)** The Bacteroidetes/Firmicutes (B/F) ratio of the four groups. *p < 0.05, **p < 0.01 *vs* the C group; ^#^ *p* < 0.05, ^##^ *p* < 0.01 *vs* the M group. **C**: control group; M: morphine group; M + M: morphine + methadone group; M + I: morphine + isorhynchophylline group.

Based on the results of the analysis of GM characteristics, we further evaluated the phylum-level composition of the GM of zebrafish. As shown in Figure 6, the zebrafish gut microbiome was mainly divided into five phyla, namely, Proteobacteria, Fusobacteria, Firmicutes, Bacteroidetes, and Actinobacteria. Compared with the control group, there was no significant change in the proportion of Proteobacteria, Bacteroidetes, Actinobacteria, or Fusobacteria in the intestinal microbiome of morphine-treated zebrafish; however, there was a significant increase in the proportion of Firmicutes after morphine administration. We further analyzed the Bacteroidetes/Firmicutes (B/F) ratio, which is considered to be closely related to intestinal microbial composition and disease occurrence. We found that the B/F ratio was significantly increased in the M group compared with that in the controls. Importantly, these changes could be mitigated by isorhynchophylline administration.

### Predicted Function Analysis of the Microbiome of C, M, M + M, and M + I-Treated Zebrafish

**FIGURE 7:**
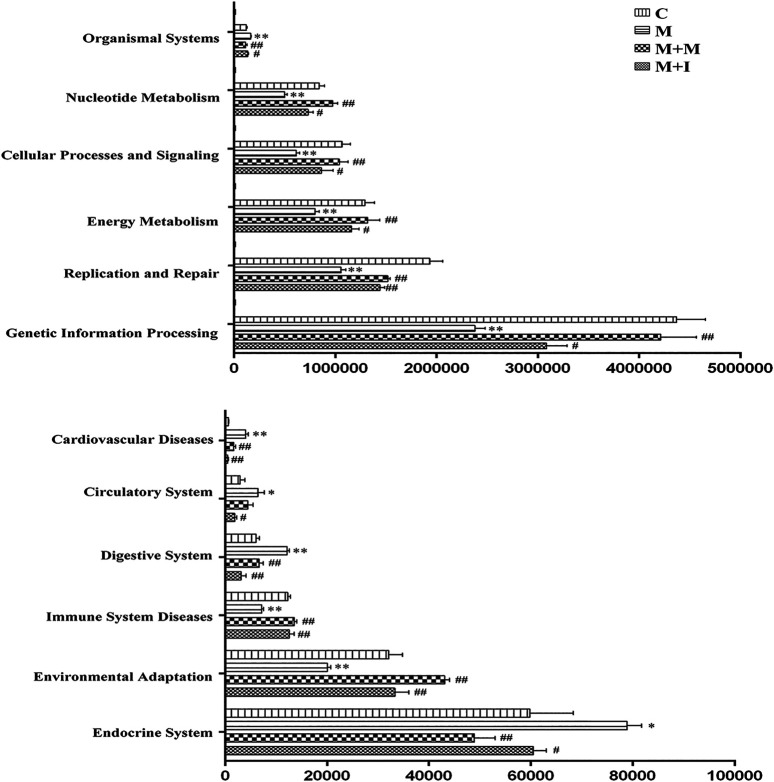
Differentially regulated functional pathways based on KEGG enrichment analysis inferred from 16S rRNA gene sequences using PICRUSt. *p < 0.05, **p < 0.01 *vs* the control (C) group; ^#^ *p* < 0.05, ^##^ *p* < 0.01 *vs* the M group.

The KEGG database is an important functional database for gene annotation. Genes can be projected into the KEGG PATHWAY database to reveal interactions with other genes that may influence the health of the host. Pathway-enrichment analysis revealed that five pathways (“Organismal Systems,” “Cardiovascular disease,” “Digestive Systems,” “Endocrine System,” and “Circulatory System”) were upregulated, while seven pathways (“Nucleotide Metabolism,” “Cellular Processes and Signaling,” “Energy Metabolism,” “Replication and Repair,” “Genetic Information Processing,” “Immune System Diseases” and “Environmental Adaption”) were downregulated in the microbiota of morphine-treated zebrafish when compared with the control group (Figure 7).

### Changes in MGBA-Related mRNA Expression in Morphine-dependent Zebrafish

**FIGURE 8:**
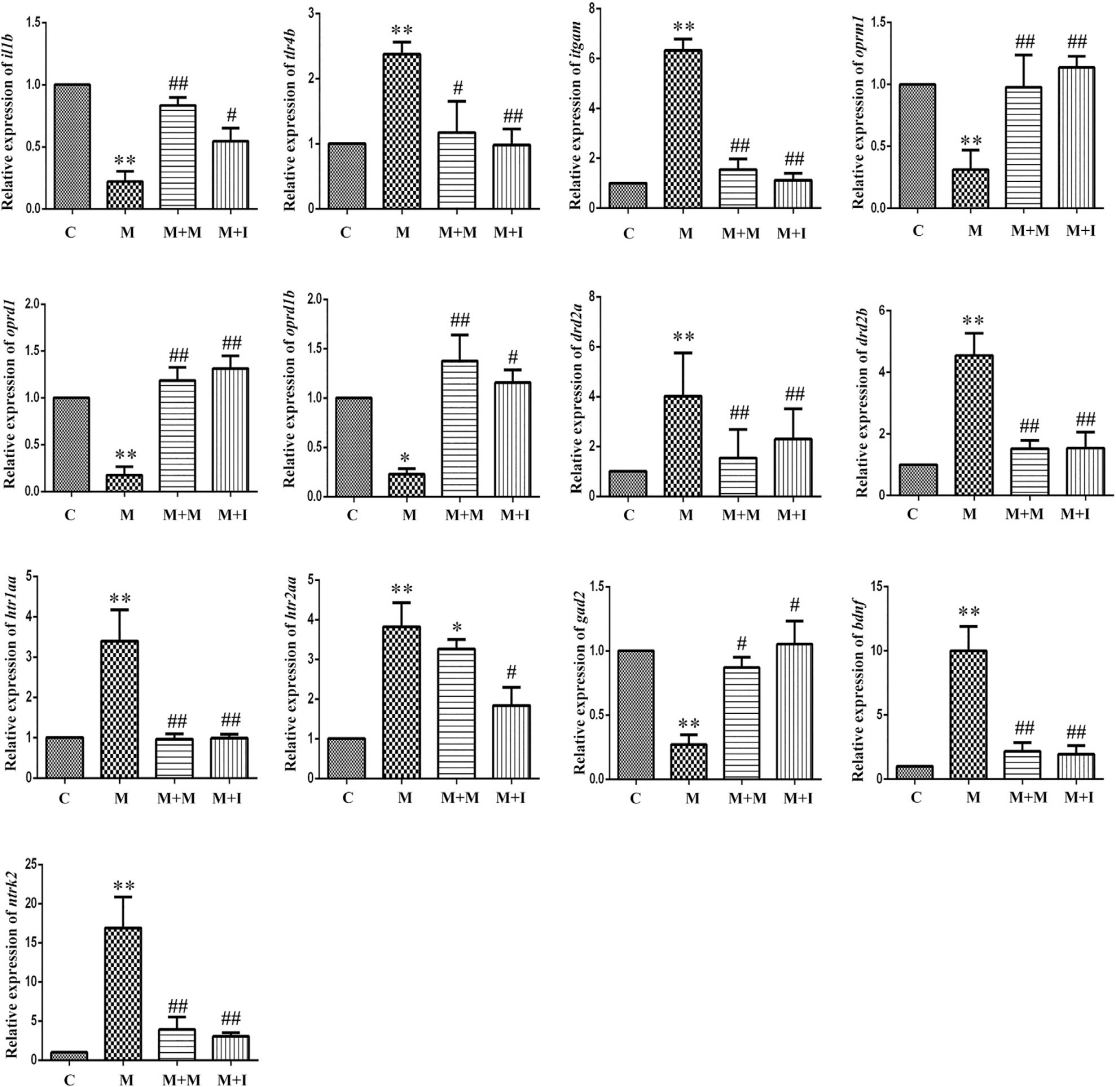
qPCR analysis of *il1b*, *tlr4b*, *itgam*, *oprm1*, *oprd1*, *oprd1b*, *gad2*, *drd2a*, *drd2b*, *htr1aa*, *htr2aa*, *bdnf*, and *ntrk2* expression in the zebrafish brain (*n* = 3). *p < 0.05, **p < 0.01 *vs* the C group; ^#^ *p* < 0.05, ^##^ *p* < 0.01 *vs* the M group. C: control group; M: morphine group; M + M: morphine + methadone group; M + I: morphine + isorhynchophylline group.

**FIGURE 9:**
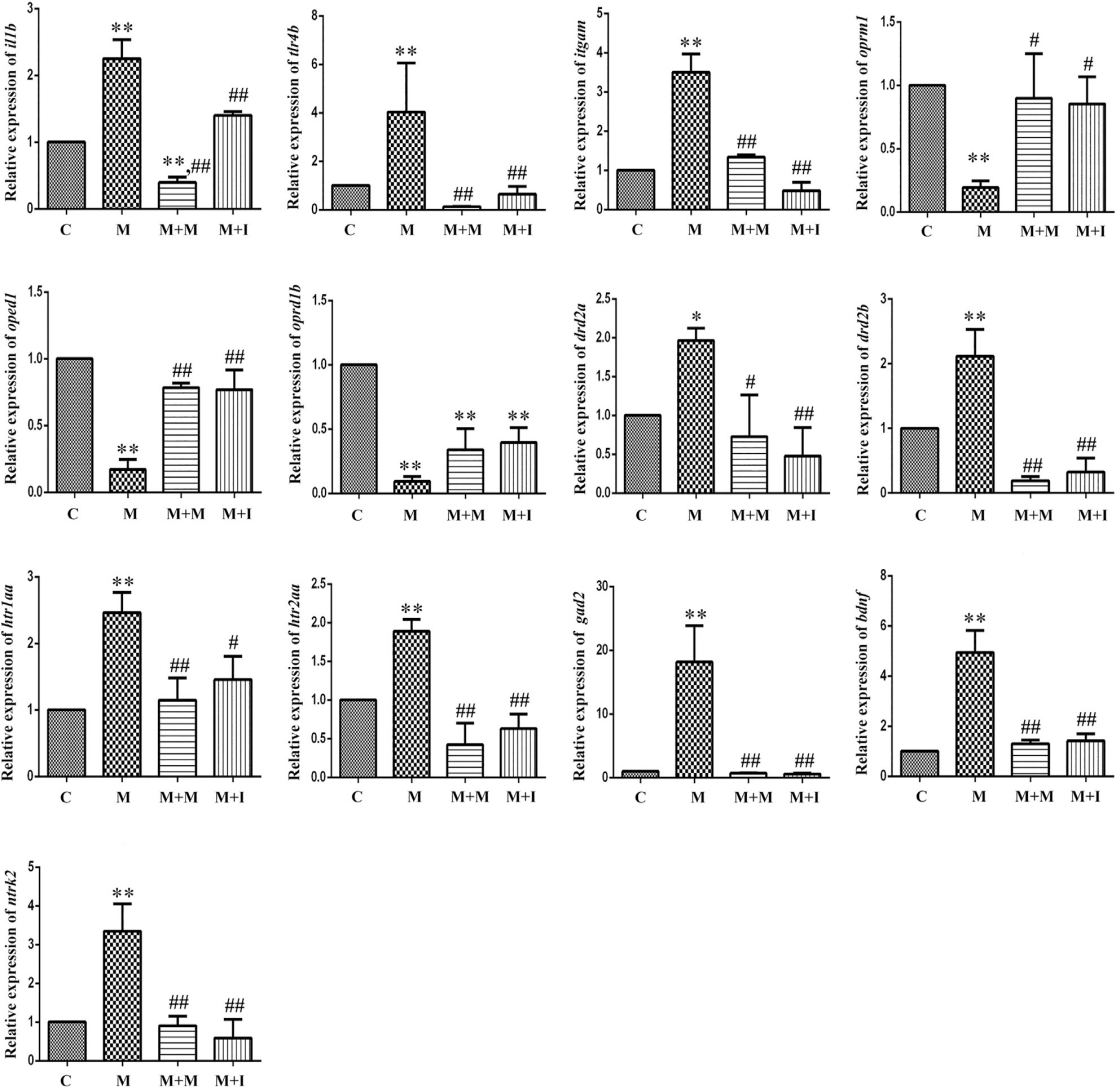
qPCR analysis of *il1b*, *tlr4b*, *itgam*, *oprm1*, *oprd1*, *oprd1b*, *gad2*, *drd2a*, *drd2b*, *htr1aa*, *htr2aa*, *bdnf*, and *ntrk2* expression in the zebrafish gut (*n* = 3). *p < 0.05, **p < 0.01 *vs* the C group; ^#^ *p* < 0.05, ^##^ *p* < 0.01 *vs* the M group. C: control group; M: morphine group; M + M: morphine + methadone group; M + I: morphine + isorhynchophylline group.

The MGBA functions primarily through endocrine, neural, immune-related, and metabolic pathways. To study how isorhynchophylline affects intestinal flora, we examined the expression of MGBA-related genes in the zebrafish brain and gut. As shown in Figures 8, 9, compared with the control group, the mRNA expression of *il1b* in the brains of zebrafish from the M group was significantly decreased, whereas it was significantly increased in the gut. Compared with the control group, the mRNA expression levels of *tlr4b* and *itgam* in the M group were significantly increased; however, these changes were reversed with isorhynchophylline treatment. Compared with that of the control group, the mRNA expression levels of *oprm1*, *oprd1/dor1*, and *oprd1b*/*dor2* were significantly decreased in the M group, whereas the mRNA expression of the three opioid receptors was significantly increased with isorhynchophylline treatment. Compared with the control group, the mRNA expression of *gad2* was significantly downregulated in the M group, while that of other neurotransmitter receptors (*drd2a*, *drd2b*, *htr1aa*, and *htr2aa*) was significantly upregulated; however, treatment with isorhynchophylline inhibited these changes. Compared with the control group, the mRNA expression levels of *bdnf* and *ntrk2* were significantly increased in the morphine treatment group, but this was reversed with administration of isorhynchophylline.

### Isorhynchophylline Cannot Inhibit Morphine-Induced CPP Responses Following Antibiotic Treatment

**FIGURE 10:**
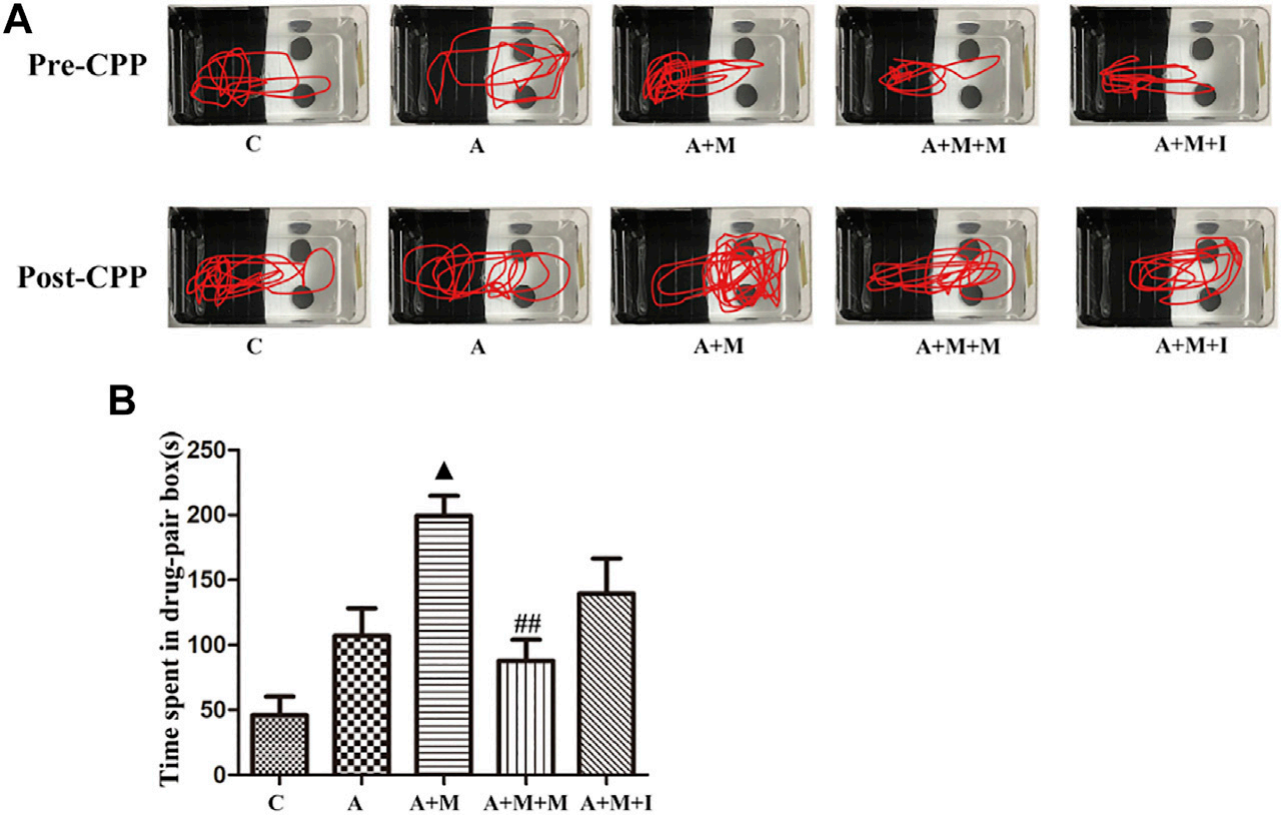
Isorhynchophylline cannot improve morphine-induced CPP responses following antibiotic treatment. **(A)** Road maps of zebrafish in the CPP box. **(B)** Time spent in the drug-paired compartment post-CPP (*n* = 8). *p < 0.05, **p < 0.01 *vs* the C group; ^#^ *p* < 0.05, ^##^ *p* < 0.01 *vs* the A + M group; ^▲^ *p* < 0.05, ^▲▲^ *p* < 0.01 *vs* the A group.

To investigate the effects of GM dysbiosis on morphine-induced zebrafish behavior, we pretreated zebrafish with antibiotics before performing morphine-dependent CPP training, followed by CPP testing. Compared with the control group, after simultaneous administration of multiple antibiotics, the residence time of antibiotic-treated zebrafish (group A) in the medicine box did not change significantly; however, when compared with the A group, the residence time of the zebrafish from the A + M group in the drug-paired compartment was noticeably increased. The residence time of the zebrafish from the A + M + I group in the drug-paired compartment was not significantly changed when compared with that of zebrafish from the A + M group (Figure 10).

C: control group; A: antibiotic group; A + M: antibiotic + morphine group; A + M + M: antibiotic + morphine + methadone group; A + M + I: antibiotic + morphine + isorhynchophylline group.

### Antibiotic Treatment Inhibited the Ameliorative Effects of Isorhynchophylline on the Morphine-Induced Changes in the Composition of the Zebrafish Gut Microbiota

**FIGURE 11:**
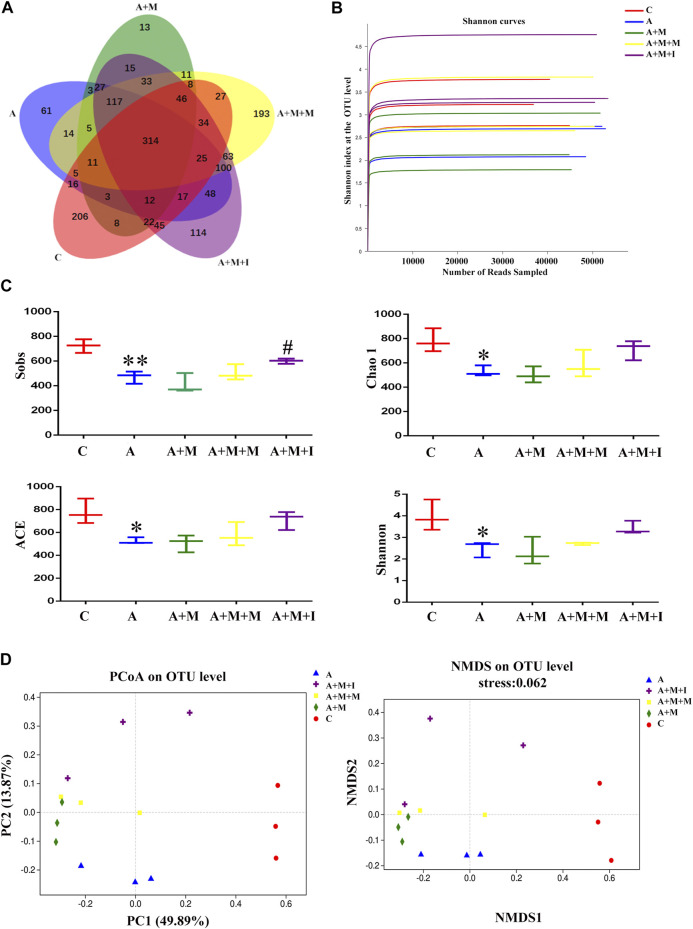
Comparison of community structure among the fecal microbiota of the C, A, A + M, A + M + M, and A + M + I groups. **(A)** Venn diagram illustrating the overlap of the OTUs identified in the fecal microbiota of the five groups. **(B)** Shannon curves among the five groups. **(C)** The alpha diversity of the fecal microbiome among the five groups based on Sobs, Chao, ACE, and Shannon index values. **(D)** The beta diversity of the fecal microbiome among the five groups based on principal coordinate analysis (PCoA) and nonmetric multidimensional scaling (NMDS). *p < 0.05, **p < 0.01 *vs* the C group; ^#^ *p* < 0.05, ^##^ *p* < 0.01 *vs* the A + M group; ^▲^ *p* < 0.05, ^▲▲^ *p* < 0.01 *vs* the A group. **C**: control group; **A**: antibiotic group; A + M: antibiotic + morphine group; A + M + M: antibiotic + morphine + methadone group; A + M + I: antibiotic + morphine + isorhynchophylline group.

To assess the changes occurring in the GM of zebrafish after antibiotic exposure, we performed massively parallel sequencing using the Miseq platform. We obtained 1,865,580 optimized sequences among all the samples with an average length of 441.34 bp. As shown by the Venn diagram (Figure 11A), a total of 1,616 OTUs were identified, 314 of which were shared by the five groups. A total of 206 OTUs were unique to the control group, 61 to the A group, 13 to the A + M group, 193 to the A + M + M group, and 114 to the A + M + I group. The Shannon curve was saturated, indicating that sufficient sequencing depth was obtained (Figure 11B). The alpha diversity index (Sobs, Chao 1, ACE, and Shannon indices) values showed that, compared with the control group, the diversity and abundance of GM in zebrafish from the A group were significantly decreased, while those of zebrafish from the A + M, A + M + M, and A + M + I groups were not significantly different (Figure 11C). In addition, we applied the Bray–Curtis distance algorithm to the PCoA and NMDS at the OTU level (Figure 11D). The PCoA and NMDS of Bray–Curtis distances showed that the composition of intestinal microbial communities differed significantly among the C, A, A + M, A + M + M, and A + M + I groups.

### Isorhynchophylline Cannot Ameliorate the Morphine Dependence-Induced Changes in the Gut Microbiota in Zebrafish After Antibiotic Treatment

**FIGURE 12:**
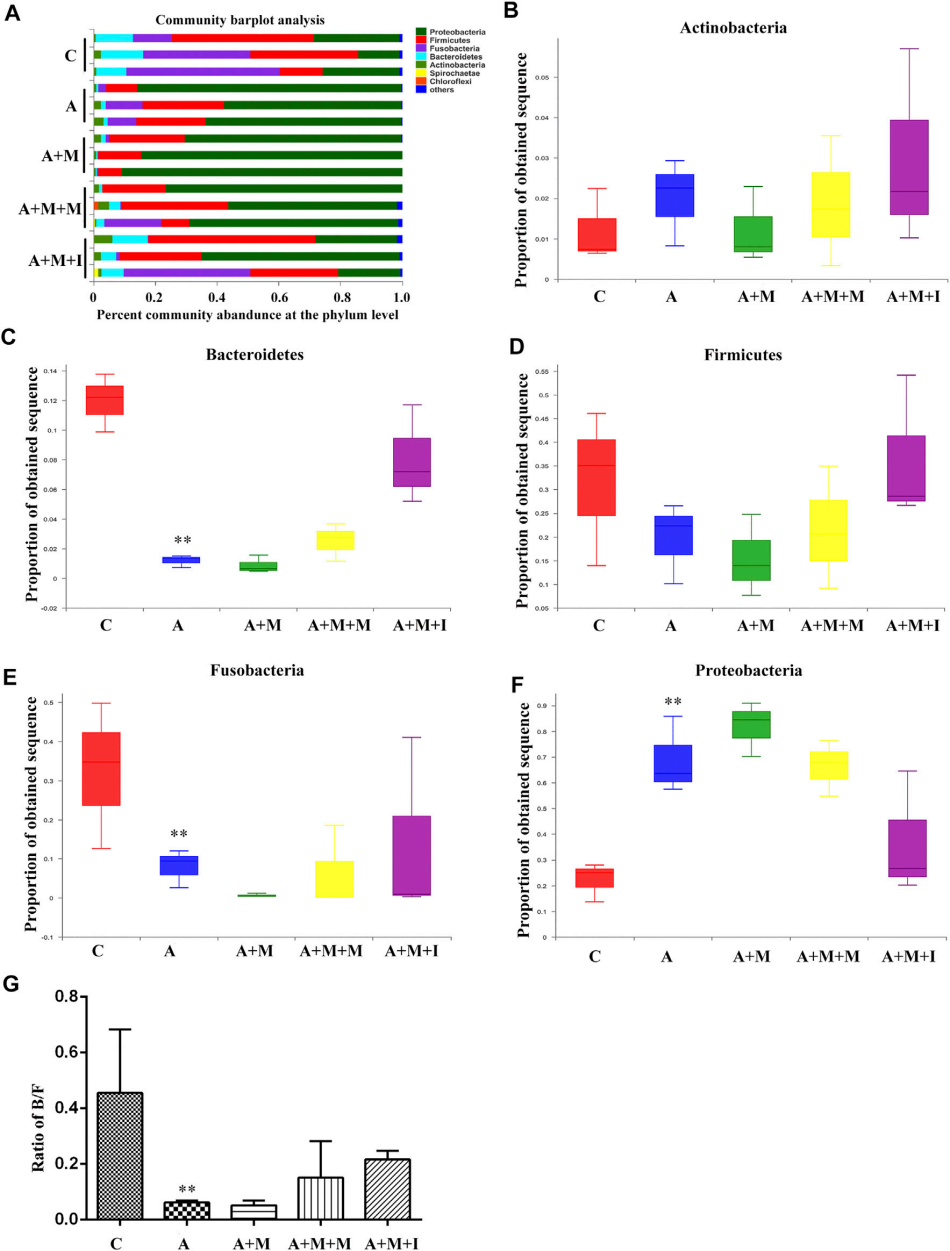
Comparison of species composition among the fecal microbiota of the C, A, A + M, A + M + M, and A + M + I groups at the phylum level. **(A)** Relative abundances of the phyla present in samples from the five groups. Percent of **(B)** Actinobacteria abundance **(C)** Bacteroidetes abundance **(D)** Firmicutes abundance **(E)** Fusobacteria abundance, and **(F)** Proteobacteria abundance at the phylum level among the five groups. **(G)** The Bacteroidetes/Firmicutes (B/F) ratio of the five groups. *p < 0.05, **p < 0.01 *vs* the C group; ^#^ *p* < 0.05, ^##^ *p* < 0.01 *vs* the A + M group; ^▲^ *p* < 0.05, ^▲▲^ *p* < 0.01 *vs* the A group. C: control group; A: antibiotic group; A + M: antibiotic + morphine group; A + M + M: antibiotic + morphine + methadone group; A + M + I: antibiotic + morphine + isorhynchophylline group.

Based on the antibiotic treatment-induced changes in the characteristics of the GM of zebrafish, we further analyzed the composition of the zebrafish gut flora. As shown in Figure 12, after treatment with antibiotics, there was no significant change in the composition of the dominant microbiota of the zebrafish gut (Proteobacteria, Fusobacteria, Firmicutes, Bacteroidetes, and Actinobacteria); however, the proportion of the dominant flora was greatly changed. After antibiotic administration, the proportions of Bacteroidetes and Fusobacteria in the zebrafish intestine were significantly downregulated, while that of Firmicutes was upregulated. Although there was no significant difference in the B/F ratio among the antibiotic-treated groups, significant differences were observed between the groups treated or not with antibiotics.

### Predicted Function Analysis of the Microbiomes of Zebrafish From the C, A, A + M, A + M + M, and A + M + I Treatment Groups

**FIGURE 13:**
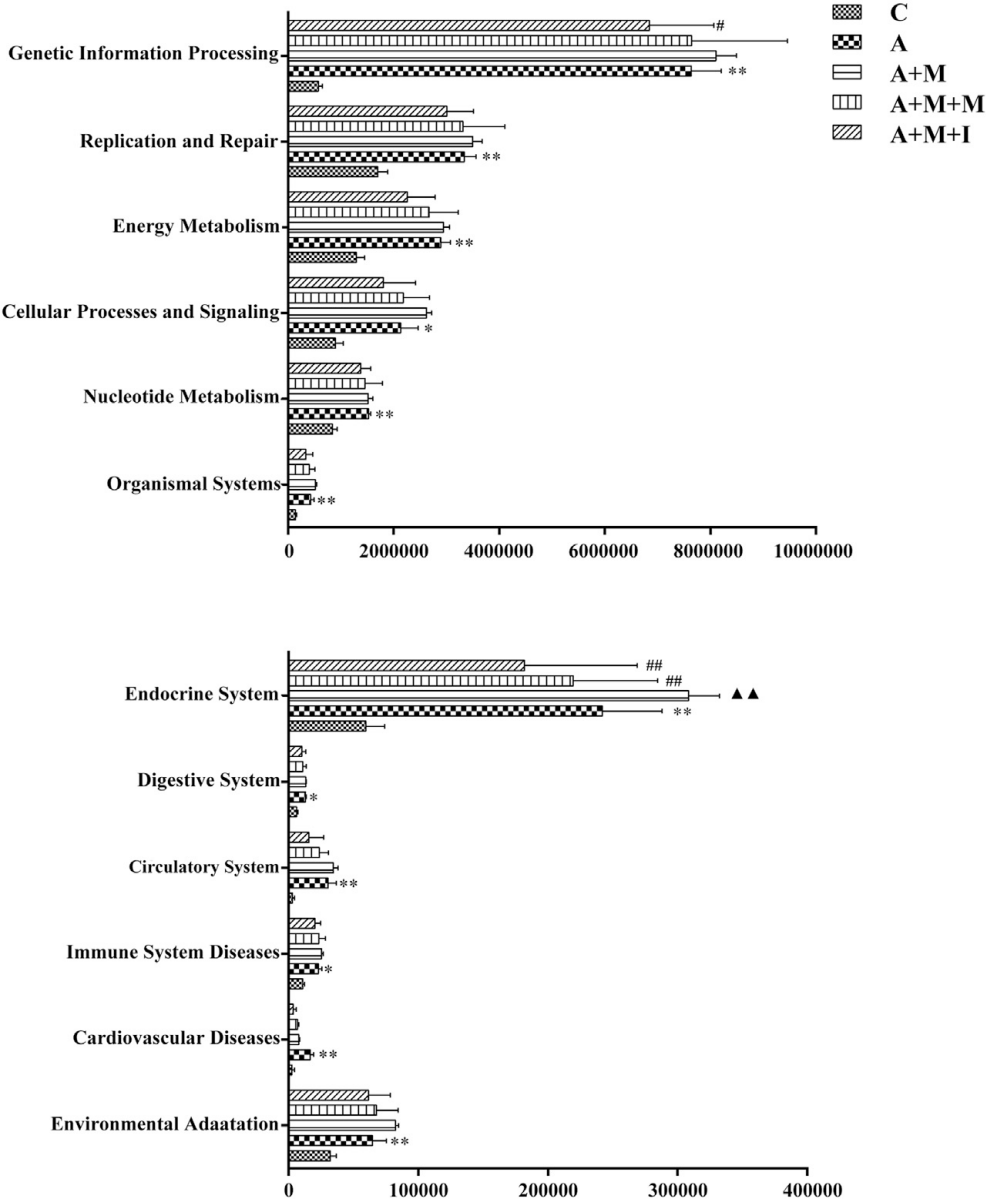
Differentially regulated functional pathways based on KEGG enrichment analysis inferred from 16S rRNA gene sequencing using PICRUSt. *p < 0.05, **p < 0.01 *vs* the control (C) group; ^#^ *p* < 0.05, ^##^ *p* < 0.01 *vs* the A + M treatment group; ^▲^ *p* < 0.05, ^▲▲^ *p* < 0.01 *vs* the antibiotic (A) treatment group.

To further reveal how GM imbalance affects morphine dependence in zebrafish and the intervention effects of isorhynchophylline, KEGG functional orthologs were predicted with PICRUSt. Figure 13 shows the differentially regulated metabolic pathways based on KEGG enrichment analysis. Pathway-enrichment analysis revealed that, compared with the control group, 13 pathways (“Neurodegenerative Diseases,” “Human disease,” “Glycan Biosynthesis and Metabolism,” “Genetic Information Processing” “Energy Metabolism,” “Lipid Metabolism,” “Cardiovascular Diseases,” “Excretory System,” “Metabolic Diseases,” “Cancers,” “Endocrine System,” “Cell Growth and Death,” and “Infectious disease”) were upregulated after antibiotic administration. However, no significant differences were found in the regulation of these metabolic pathways in the gut of zebrafish from all the treatment groups after antibiotic administration.

### Changes in MGBA-Related mRNA Expression in Morphine-Dependent Zebrafish After Antibiotic Treatment

**FIGURE 14:**
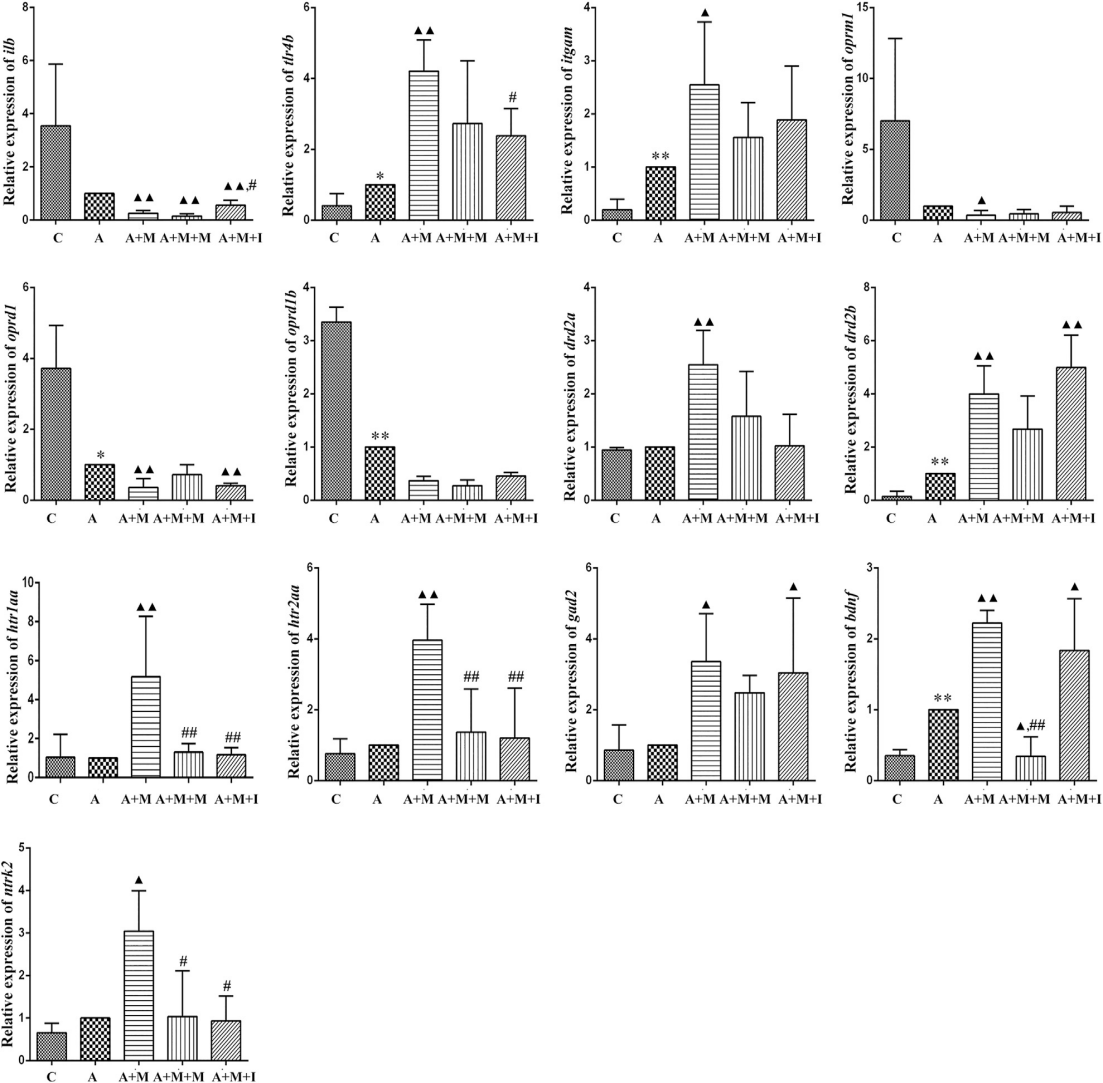
qPCR analysis of *il1b*, *tlr4b*, *itgam*, *oprm1*, *oprd1*, *oprd1b*, *gad2*, *drd2a*, *drd2b*, *htr1aa*, *htr2aa*, *bdnf*, and *ntrk2* expression in the zebrafish brain (*n* = 3). *p < 0.05, **p < 0.01 *vs* the C group; ^#^ *p* < 0.05, ^##^ *p* < 0.01 *vs* the A + M group; ^▲^ *p* < 0.05, ^▲▲^ *p* < 0.01 *vs* the A group. C: control group; A: antibiotic group; A + M: antibiotic + morphine group; A + M + M: antibiotic + morphine + methadone group; A + M + I: antibiotic + morphine + isorhynchophylline group.

**FIGURE 15:**
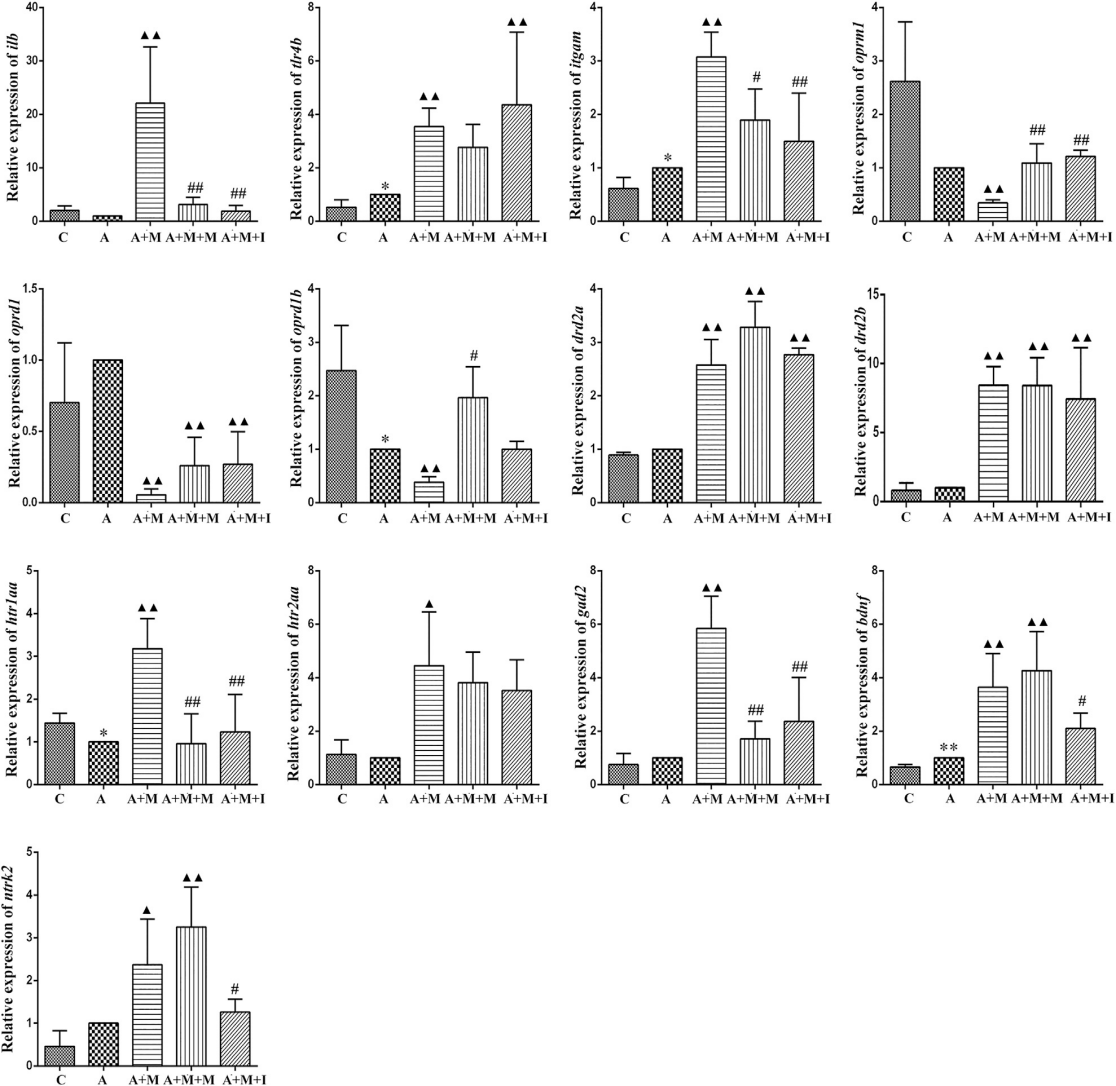
qPCR analysis of *il1b*, *tlr4b*, *itgam*, *oprm1*, *oprd1*, *oprd1b*, *gad2*, *drd2a*, *drd2b*, *htr1aa*, *htr2aa*, *bdnf*, and *ntrk2* expression in the zebrafish gut (*n* = 3). *p < 0.05, **p < 0.01 *vs* the control C group; ^#^ *p* < 0.05, ^##^ *p* < 0.01 *vs* the A + M group; ^▲^ *p* < 0.05, ^▲▲^ *p* < 0.01 *vs* the A group. C: control group; A: antibiotic group; A + M: antibiotic + morphine group; A + M + M: antibiotic + morphine + methadone group; A + M + I: antibiotic + morphine + isorhynchophylline group.

To evaluate how GM affects morphine dependence and drug intervention, we examined the expression of MGBA-related genes in the zebrafish brain and gut after antibiotic treatment. As shown in Figures 14, 15, compared with the A group, the mRNA expression of *il1b* in the brain of zebrafish in the A + M and A + M + I groups was significantly decreased, while that in the gut of fish from the A + M group was significantly increased. Compared with the A + M group, the mRNA expression of *il1b* in the gut of zebrafish in the A + M + I group was significantly decreased. After antibiotic treatment, the mRNA expression of *tlr4b* and *itgam* in zebrafish increased significantly. Compared with the A group, the mRNA expression levels of *tlr4b* in the A + M group and *itgam* in the A + M group were significantly increased. Furthermore, after treatment with isorhynchophylline, the mRNA expression of *tlr4b* in the zebrafish brain and that of *itgam* in the zebrafish gut were significantly decreased. After treatment with antibiotics, the mRNA expression of *oprd1* and *oprd1b* in zebrafish decreased significantly. Compared with the A group, the mRNA expression levels of *oprm1*, *oprd1*, and *oprd1b* were significantly decreased in the A + M treatment group; however, isorhynchophylline administration inhibited the changes in the mRNA levels of *oprm1* and *oprd1* in the zebrafish gut and *oprd1* in the zebrafish brain. Following antibiotic treatment, the mRNA expression of *drd2b* in the zebrafish brain increased significantly. Compared with the A group, the mRNA expression levels of *drd2b* and *gad2* in the brain of fish from the A + M + I group and *drd2a* and *drd2b* in the gut of fish from the A + M + I group were significantly upregulated. Compared with the A + M group, the mRNA expression of *htr1aa* and *htr2aa* in the brain and *htr1aa* and *gad2* in the gut of A + M + I-treated zebrafish were markedly decreased. After treatment with antibiotics, the mRNA expression of *bdnf* in zebrafish increased significantly. Compared with the A group, the mRNA expression of *bdnf* and *ntrk2* in the A + M group was significantly increased in both the brain and the gut, while the mRNA expression of *bdnf* in the brains of fish from the A + M + I group was significantly increased. Compared with the A + M group, the mRNA expression levels of *bdnf* and *ntrk2* in the gut of zebrafish from the A + M + I group were significantly lower.

## Discussion

To the best of our knowledge, this is the first study characterizing the morphine-induced changes in MGBA-related mRNA expression in the brain and intestine of morphine-dependent zebrafish, as well as in activated BV2 microglial cells. Specifically, after observing the effect of morphine on the GM of zebrafish, multiple antibiotics were administered simultaneously to analyze how GM dysbiosis affects morphine addiction using zebrafish as a model. Moreover, the effects of isorhynchophylline on morphine dependence were investigated based on changes in the expression of MGBA-related genes both *in vivo* and *in vitro*. We also studied the effects of isorhynchophylline on morphine-dependent zebrafish and the expression of MGBA-related genes under normal or disordered GM conditions. Our findings provide a novel framework for understanding the mechanisms of morphine addiction through the MGBA and may provide new TCM-based therapeutic strategies for the prevention of drug addiction.

Neuronal cells account for approximately only 10% of the total cell number in the CNS, while glial cells comprise the remaining 90%. Glial cells are mainly divided into microglia and astrocytes, with microglia accounting for 10–15% of the total number of cells in the CNS (Watkins et al., 2009). Microglia are the innate immune cells of the brain. Several studies have found that glial cells in the CNS can be activated by opioids such as heroin and morphine, secrete inflammatory factors, amplify neuronal excitability, and regulate the reward system, thereby contributing to opioid addiction-related behavior (Hutchinson et al., 2011; Cui et al., 2014; Wardill et al., 2015). In this study, BV2 cells were activated following morphine administration, showing an increase in the expression of AIF1, a marker protein for activated BV2 cells.

The time that animals spend in locations where they have access to a medicine can be used to better understand their psychological dependence on that medicine (Tzschentke, 2007). Irrespective of whether the intestinal microflora of zebrafish was disturbed by antibiotic administration, after being given morphine and after CPP training, their residence time in the drug-paired compartment increased significantly. However, the residence time of the morphine-treated zebrafish in the drug-paired compartment was significantly reduced after isorhynchophylline administration, indicating that isorhynchophylline can effectively inhibit morphine-induced place preference in zebrafish. After disruption of the zebrafish GM with antibiotic administration, the residence time of zebrafish from the A + M + I group in the drug-paired compartment was not significantly different from that of fish from the A + M group, indicating that antibiotic treatment can inhibit the ameliorative effects of isorhynchophylline on zebrafish GM dysbiosis.

Studies have shown that GF mice display increased motor activity and reduced anxiety-like behavior when compared with specific-pathogen-free SPF mice. Increasing evidence has indicated that the GM regulates brain function, including learning and memory. Studies have also shown that, compared with SPF animals, GF mice exhibit both reduced basal anxiety-like behavior and increased resistance to LPS-induced depressive-like and anhedonia-like behaviors (Campos et al., 2016). Moreover, rats chronically treated with antibiotics show deficits in spatial memory and a greater display of depressive-like behaviors in the forced swim test (Hoban et al., 2016). While mice were found to exhibit more anxious behavior in a mouse model of dextran sodium sulfate-induced enteritis (Bercik et al., 2011). However, relatively few studies have investigated the link between drug addiction and GM imbalance, which requires further in-depth research.

The GM is closely related to numerous aspects of normal human and animal physiology. These intestinal microorganisms are generally in a dynamic equilibrium, and maintain a mutually beneficial symbiotic relationship with their host. However, the GM is prone to imbalance owing to host growth and development or changes in host diet. In this study, we used 16S rRNA sequencing to analyze the composition of the intestinal microbiota of zebrafish, and assessed the effects of isorhynchophylline on the intestinal flora of morphine-dependent zebrafish. Shannon, Sobs, Chao 1, and ACE indices are commonly used to measure gut microflora community richness and diversity. Several studies have shown that GM community richness and diversity in patients with depression and schizophrenia are significantly lower than those in healthy controls (Zheng et al., 2016; Zheng et al., 2019). In our study, the community richness and diversity of the GM of morphine-treated zebrafish decreased significantly when compared with non-treated controls, but this effect could be reversed by isorhynchophylline administration. The community richness and diversity of the GM of antibiotic-administered zebrafish also showed a significant decrease when compared with the control group. However, no significant difference was recorded in community richness and diversity in the GM of zebrafish treated with both morphine and antibiotics, possibly because antibiotic administration disturbed the zebrafish gut flora, which may have masked the effect of morphine treatment. Importantly, this result echoes that of the CPP test, indicating the interdependence of the GM and CNS.

The composition of the intestinal microflora is complex. However, Bacteroidetes and Firmicutes are known to be the predominant GM bacterial phyla, the ratio of which is considered to be closely related to disease occurrence. In this study, a reduction in the comparative abundance of Firmicutes was observed in morphine-treated animals, which increased the B/F ratio. Similar B/F ratio changes are observed in autistic patients (Zhang et al., 2018). We further compared the B/F ratios among the GM of all the treatment groups before and after antibiotic use, and found that the overall B/F ratios of the four groups after antibiotic treatment were significantly lower than before treatment, possibly explaining why no significant difference was observed in the B/F ratio of the zebrafish GM after antibiotic treatment. The results indicated that concomitant administration of multiple antibiotics changed the composition and proportion of the species that comprise the intestinal flora. Pathway-enrichment analysis showed that morphine treatment upregulated five pathways and decreased seven metabolic pathways, eight of which (Cardiovascular disease, Endocrine System, Circulatory System, Cellular Processing and Signaling, Energy metabolism, Genetic Information Processing, Immune System disease, and Environmental Adaption) are closely associated with morphine use (Tung et al., 2015; Gimenez-Mila et al., 2016; Balyan et al., 2017; Hakimian et al., 2017; Park et al., 2017; de Vries et al., 2019; Denny and Unterwald, 2019; Mutua et al., 2019). In accordance with the results for GM composition, no differences were observed in the regulation of the above metabolic pathways in the GM of A-, A + M-, A + M + M-, and A + M + I-treated zebrafish after antibiotic treatment. Importantly, the morphine-induced changes in GM composition and metabolic pathway regulation were ameliorated with administration of isorhynchophylline. However, and consistent with the behavioral findings, isorhynchophylline treatment did not reverse the morphine-induced changes in the intestinal flora of zebrafish following antibiotic treatment. Combined, the results showed not only that the drugs (morphine and isorhynchophylline) could change the composition of the intestinal flora, but that the changes in the composition of the intestinal flora could also affect the pharmacodynamics of the drugs.

Under normal physiological conditions, the intestinal flora maintains a dynamic balance, which can help protect and maintain the health of the host. Changes in the intestinal flora can lead to disturbances in the perception, transmission, and reaction of intestinal epithelial cells to intestinal bacterial-derived signals, as well as intestinal mucosal damage, which can eventually induce a disordered immune response (Goto and Kiyono, 2012). In the present study, the expression of *il1b* in the zebrafish brain was significantly decreased with morphine administration and significantly increased following administration of isorhynchophylline. Interestingly, *Il1b* expression in the zebrafish gut and BV2 cells was significantly increased after exposure to morphine. In the zebrafish, this may have resulted from morphine intraperitoneal injection directly affecting the zebrafish intestinal tract, leading to dysregulation of the intestinal flora and increased expression of inflammatory cytokines in the intestine. Gut microbiota imbalance has been shown to lead to the production of a variety of cytokines and endotoxins, excitation of the HPA axis, and promotion of catecholamine release, thereby affecting movement and secretion of the gastrointestinal tract. Several studies have found that TLR4 inhibitors can suppress the morphine-induced activation of the microglial TLR4 signaling pathway, as well as withdrawal symptoms and CPP behavior, and reward behavior was shown to be absent in TLR4-knockout mice (Terashvili et al., 2007; Hutchinson et al., 2010; Hutchinson et al., 2012). In the morphine-treated zebrafish and morphine-induced BV2 microglial cells, the mRNA expression of *Tlr4*, *Itgam*, and *Nos2* was significantly increased, and these changes were partially reversed following treatment with isorhynchophylline. The effects of morphine on immune inflammation correspond to the immune system disease pathway identified in the KEGG pathway analysis of the GM (Figures 8, 13), which confirms the relationship between GM and morphine addiction.

Both mu (Oprm) and delta opioid receptors (Oprd) are known to be involved in opioid dependence (Shippenberg et al., 2009). Studies have shown that, during morphine addiction, the expression of both mu and delta opioid receptor-related genes are downregulated in the rat brain. Additionally, both mu and delta opioid receptor agonists can influence CPP in rats (Tempel et al., 1988; Noble et al., 2000). Consistent with the results of previous studies, we found that the expression of genes coding for Oprms and Oprds was significantly downregulated in morphine-treated zebrafish and BV2 cells, irrespective of whether intestinal disorders had been induced with antibiotic treatment. Isorhynchophylline could inhibit the morphine-induced downregulation of the expression of genes coding for Oprms and Oprds in both zebrafish and BV2 cells. When the intestinal flora was disrupted by antibiotic treatment, isorhynchophylline could no longer ameliorate the morphine-induced effects, indicating that there is an interaction between opioid receptors, morphine addiction, and intestinal flora.

Most neurotransmitters are highly abundant in the brain and enteric nervous system. As the brain is the focus of drug addiction and the intestine is an important member of the “Brain-Gut Axis,” substantial attention has been paid to the neurotransmitters in these two tissues. Neurotransmitters are involved in numerous biological processes, such as learning, memory, and metabolism (Bergquist et al., 2002; Cai et al., 2010; He et al., 2016). Dopamine (DA) receptors are classified into types D1 (including D1 and D5 receptors) and D2 (including D2, D3, and D4 receptors), with D2 receptors playing a more important role in opioid addiction (Wood, 2008; Dai et al., 2016). Studies have demonstrated that CPP was inhibited in dopamine D2 receptor-deficient mice following morphine administration. In addition, D2 receptor antagonists can effectively reduce morphine tolerance and drug-seeking behavior in rats (Ozdemir et al., 2013). An increasing number of studies have confirmed that the 5-HT system is closely related to drug addiction. Activation of 5-HT receptors and release of neurotransmitters can affect many effects and neuroexcitatory conduction processes involved in drug addiction (Weitemier and Murphy, 2009; Gomez-Milanes et al., 2012). Moreover, morphine can act on opioid receptors in GABA neurons, thereby affecting the release of 5-HT and DA (Tao and Auerbach, 2002). Glutamic acid decarboxylase (GAD), the GABA-synthesizing enzyme, is thought to be associated with morphine addiction. Long-lasting changes in *GAD67* mRNA expression have been found following chronic drug exposure (nicotine, morphine, and amphetamine) (Lindefors et al., 1992; Cadoni and Di Chiara, 1999; Cadoni and Di Chiara, 2000; Cadoni et al., 2000; Cadoni et al., 2003). In this study, the expression of various neurotransmitter-related genes in zebrafish and BV2 cells was significantly upregulated after exposure to morphine. Similar to the opioid receptor test, isorhynchophylline can inhibit morphine-induced changes in neurotransmitter-related gene expression when the zebrafish GM is normal, but not when antibiotics are used.

Brain-derived neurotrophic factor (BDNF) is a neurotrophic protein in the brain, and neurotrophic tyrosine receptor kinase 2 (NTRK2) is a high-affinity receptor for BDNF. Numerous studies have shown that the normal role of BDNF/NTRK2 signaling is closely associated with advanced cognitive functions of learning and memory (Yamada and Nabeshima, 2003). In addition to being widely expressed in the central nervous system, BDNF is also expressed in a large number of peripheral tissues, including the intestine. Early studies reported extensive *BDNF* mRNA expression in the intestinal epithelium, ring muscles, and myenteric plexus (Acosta et al., 2015; Agrawal et al., 2017). Moreover, in animal models of intestinal inflammation, changes in bacterial flora were shown to reduce the expression of *BDNF* mRNA in the hippocampus (Bercik et al., 2010). This indicates that the GM is connected to the CNS through BDNF. Morphine use has been shown to increase the expression of BDNF and NTRK2 in the brain (Numan et al., 1998; Akbarian et al., 2002; Bolanos and Nestler, 2004; Vargas-Perez et al., 2009; Koo et al., 2012; Mashayekhi et al., 2012; Peregud et al., 2016; Naghshvarian et al., 2017). In our study, the mRNA expression of *bndf* and *ntrk2* in zebrafish increased significantly after morphine administration, while isorhynchophylline reversed this change. This indicates that the BDNF/NTRK2 signaling pathway in the peripheral nervous system is also involved in the process of morphine addiction.

Our combined results provide seminal evidence that the effect of isorhynchophylline on morphine dependence is related to the intestinal microflora. Moreover, we found that dysbacteriosis can change morphine-dependent behavior and affect the efficacy of isorhynchophylline, which are accompanied by metabolic changes in the MGBA. Our findings provide a novel framework for understanding the mechanisms involved in TCM-derived antagonism of morphine addiction through the MGBA and may lead to new diagnostic and treatment strategies for this condition.

## Data Availability

Illumina whole-genome sequencing (WGS) and RNA-seq reads have been deposited in the NCBI sequence read archive (SRA) under the Bioproject PRJNA587320 accession number.
