# Supplementary material for: The Ameliorative Effects of Isorhynchophylline on Morphine Dependence Are Mediated Through the Microbiota-Gut-Brain Axis
Source: Front Pharmacol. 2021 Jun 8;12:526923. doi: 10.3389/fphar.2021.526923 (PMC8218633; doi:10.3389/fphar.2021.526923)
Supplement: Supplementary file 1 [file datasheet1.docx]

**Tables**

**TABLE S1** Reaction condition of PCR amplification

| Temperature | Time | Cycle |
| --- | --- | --- |
| 95℃ | 3min | 1 |
| 95℃ | 30s | 27  27  27 |
| 55℃ | 30s |  |
| 72℃ | 30s |  |
| 72℃ | 10min | 1 |

**TABLE S2** Reaction system of PCR amplification

| Reagent | Volume |
| --- | --- |
| 5 × FastPfu Buffer | 4 μL |
| dNTPs (2.5 mM) | 2 μL |
| primer (5 μM) | 0.8μL |
| FastPfu Polymerase | 0.4 μL |
| DNA (10 ng) | 12.8 μL |
| Total | 20 μL |

**TABLE S3** The PCR primer sequence of mRNA

| mRNA | PCR primer（5’-3’） |
| --- | --- |
| zf-b-actin F  zf-b-actin R  zf-il1b F | ATGGATGAGGAAATCGCTG  ATGCCAACCATCACTCCCTG  TGGACTTCGCAGCACAAAATG |
| zf-il1b R | GTTCACTTCACGCTCTTGGATG |
| zf-oprm1 F | ACGAGCTGTGCAAGATTGTG |
| zf-oprm1 R | CCGATTGCAGATGAAAGGAT |
| zf-oprd1 F | ACTATGAGAGCGTGGACCGTT |
| zf-oprd1 R | GCGGAGGAGAGGATCCAGAT |
| zf-oprd1b F | TCAGGCAAAACAATCTGCATG |
| zf-oprd1b R | CAGGATCATCAGGCCGTAGC |
| zf-htr1aa F | GGAGCCCGCCATGCGTCTT |
| zf-htr1aa R  zf-htr2aa F  zf-htr2aa R | CGTCGCGTTCCCGCTCCAA  GCATCTCTCTTACCCAATCTATCC  AACTAACTCCTCTTTGGTCGTCTC |
| zf-gad2 F | CTGCATCAGTCCCTCCTCTC |
| zf-gad2 R | AAAGTATCAGAGACCCGAGCAA |
| zf-tlr4b F | ATTCCACCGTCTTAAGGAACTGT |
| zf-tlr4b R | CAGTGAAGGTAATAGGAAAGGGAC |
| zf-drd2a F | TGGTACTCCGGAAAAGACG |
| zf-drd2a R | ATCGGGATGGGTGCATTTC |
| zf-drd2b F | CGTGCCCTTCATTATCACC |
| zf-drd2b R | TAGCGAGGATGCCAGTTTTT |
| zf-itgam F | ACGTGACGCTGTTTGTCG |
| zf-itgam R | GCCAGCAGCACAAGTCC |
| zf-bdnf F | AACATTCCGTTTACATTCTC |
| zf-bdnf R | ACAACAGCACCTTGACATAG |
| zf-ntrk2 F | GGAAAAGCAAAAACCCTGTCTAGA |
| zf-ntrk2 R | TGTAGCATCACTTCCTGCCATT |
| m-Itgam-F | CTTCCAGGGCAGGAGTCGTA |
| m-Itgam-R | CCCACAATGAGTGGTACAGGATT |
| m-NOS2-F | CCCTTCCGAAGTTTCTGGCAGCAGC |
| m-NOS2-R | CCCTTCCGAAGTTTCTGGCAGCAGC |
| m-β-Actin F  m-β-Actin R  m-Il1b F | TTCCTTCTTGGGTATGGAAT  GAGCAATGATCTTGATCTTC  ATGCCACCTTTTGACAGTGATG |
| m-Il1b R | TGATGTGCTGCTGCGAGATT |
| m-Tlr4 F | GGCTCATTCACTCACTAACGGG |
| m-Tlr4 R | CACTGTCACAGGAGGGACCA |
| m-Oprd1 F | TAGACACTCACTGGAAAGTCCTGCT |
| m-Oprd1 R | CTCTAACACAACTCTGCCCTGG |
| m-Htr2a F | GGCGATTCTGCCTGAGACTAAA |
| m-Htr2a R | ACAGCATGAGATCCAAAACGAG |
| m-Drd2 F | CCTTCTTCATCACGCACATCCT |
| m-Drd2 R | GTTCACGGCACTGTTGACATAG |
| m-Gad2 F | CCTTTCCACTTTCCAATCTCCA |
| m-Gad2 R | GCGTGTTTGTATGTTCCAGTCA |
| m-Gad1 F | CACAGGTCACCCTCGATTTTTC |
| m-Gad1 R | ATGTCAGCCATTCACCAGCTAA |
| m-Oprm1 F  m-Oprm1 R | GGTGGTCGTGGCTGTATTTATT  CAGTCTGGAAAGTGGTTTCTGG |
| m-Bdnf F | CTCGCAATGCCGAACTACC |
| m-Bdnf R | TCCTTATGAATCGCCAGCC |
| m-Ntrk2 F | CGTCACCAATCACACGGAGT |
| m-Ntrk2 R | GTGAGCGGAGATCTGTCTCTCA |
